# Supplementary material for: Interaction-induced singular Fermi surface in a high-temperature oxypnictide superconductor
Source: Sci Rep. 2015 May 21;5:10392. doi: 10.1038/srep10392 (PMC4441009; doi:10.1038/srep10392)
Supplement: Supplementary Information [file srep10392-s1.pdf]

Interaction-induced singular Fermi surface in a high-temperature oxypnictide superconductor

A. Charnukha[1], S. Thirupathaiah[1], V. B. Zabolotnyy[1], B. Büchner[1], N. D. Zhigadlo[2], B. Batlogg[2], A. N. Yaresko[3] & S. V. Borisenko[1]

[1]*Leibniz Institute for Solid State and Materials Research, IFW, D-01069 Dresden, Germany*
[2]*Laboratory for Solid State Physics, ETH Zurich, CH-8093 Zurich, Switzerland*
[3]*Max Planck Institute for Solid State Research, 70569 Stuttgart, Germany*

I. LOW-ENERGY ELECTRONIC STRUCTURE OF $SmFe_{0.92}Co_{0.08}AsO$ NEAR THE Γ POINT

In order to confirm the existence of *two* electronic bands terminating in the immediate vicinity of the Γ point but split by an amount of approximately 20 meV, as stated in the main text, we plot in Fig. S1 the distribution of the photoemission intensity in an energy-momentum cut along the Γ–M direction of the Brillouin zone as well as the second derivative of this signal taken along the momentum axis. Already the raw data in Fig. S1a clearly indicate the presence of four bands with holelike electronic dispersion around the Γ, two of which cross the Fermi level (white arrows in Fig. S1b), while the other two terminate in its immediate vicinity (white dashed lines in Fig. S1b). The splitting between the latter is ≈ 22 meV. It is much smaller than its theoretically predicted counterpart (about 80 meV, see Fig.1i of the main text and Fig. S1c). However, if one assumes that this splitting is renormalized along with the underlying band structure then its renormalization ($80/22 = 3.6$) is fully consistent with the renormalization of the hole bands at the G point (a factor of about 3.3, see main text and Fig.3 therein). Figure S1c further clearly shows that the lower two hole bands, degenerate by symmetry in the absence of the spin-orbit coupling, become split when the spin-orbit coupling is taken into account. However, this splitting is significantly smaller than that between the upper two bands and could not be resolved in our measurements.

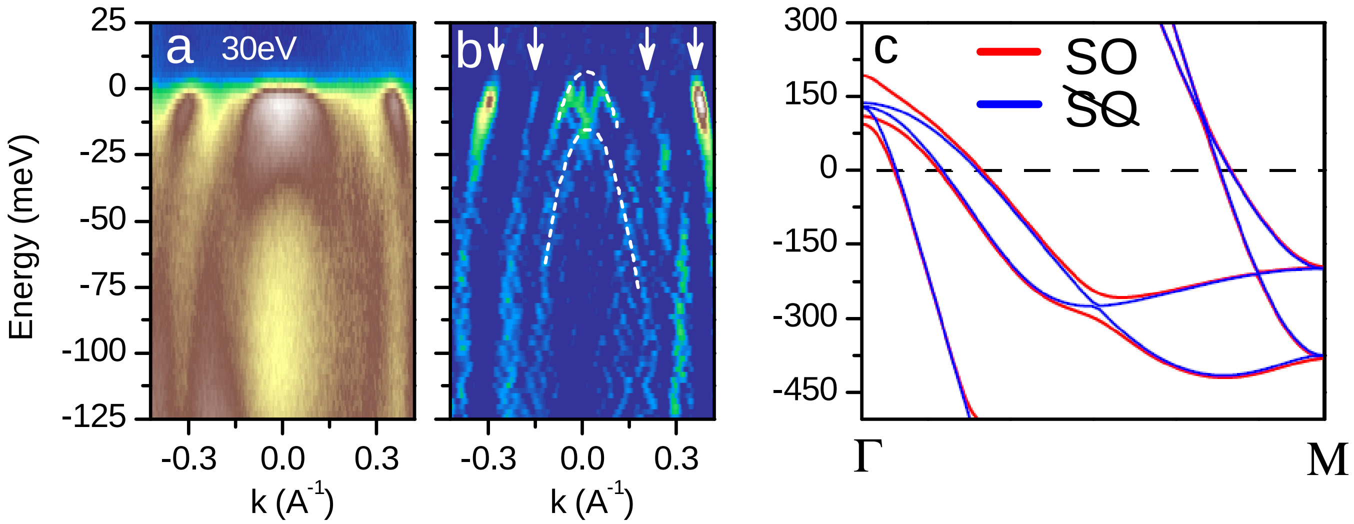

Figure S1 | Band splitting in the low-energy electronic structure of $SmFe_{0.92}Co_{0.08}AsO$ near the Γ point a Momentum-energy cut of the photoemission signal in the Γ–M direction of the Brillouin zone obtained using an incident photon energy of 30 eV. **b** Second derivative of the intensity distribution shown in panel **a** taken along the momentum axis. White arrows indicate bands clearly crossing the Fermi level, while the white dashed lines indicate those terminating in its immediate vicinity. **c** Theoretically predicted low-energy electronic structure along the Γ–M direction in the Brillouin zone, with (red lines) and without (blue lines) the spin-orbit coupling taken into account.

II. EFFECT OF SURFACE AGING ON THE LOW-ENERGY ELECTRONIC STRUCTURE OF $SmFe_{0.92}Co_{0.08}AsO$

To confirm the assignment of the band dispersions producing large circular intensity distributions near the Γ and M point in the photoemission maps of Fig.1a,b to the polar surface, we have subjected the sample surface of $SmFe_{0.92}Co_{0.08}AsO$ to intentional aging via temperature cycling between 300 and 1 K. The results of this experiment are summarized in Fig. S2. Panels S2a,c demonstrate the effect of aging on the low-energy electronic structure in the vicinity of the Γ point. While the freshly cleaved sample (Fig. S2a) clearly shows three holelike bands of comparable intensity crossing or approaching the Fermi level, in the aged material (Fig. S2c) the large outer dispersion is suppressed, strongly supporting the assignment of this band to the surface-related electronic structure in the main text. The same conclusion can be drawn from the comparison of the photon-energy–dependence of the photoemission intensity near the Fermi level before and after aging shown in Figs. S2b, d, respectively. The two outer features are very two-dimensional (have negligible k_z dispersion) and the outermost one is strongly suppressed in the aged sample, with the central bulk-related feature virtually unchanged. While the suppression of the intensity of the second outermost band is quite small, our analysis of the characteristic properties of the electronic band structure of $SmFe_{0.92}Co_{0.08}AsO$ clearly indicates that this band is incompatible with the bulk and must be extrinsic as well. Surface aging has a strong effect on the low energy electronic structure at the M point of the Brillouin zone as well. While the photoemission intensity map in the fresh sample in Fig. S2e clearly shows the presence of a large circular feature at the M point, this latter is entirely suppressed in the aged sample, as shown in Fig. S2f. This effect of aging can be seen even more clearly in the energy-momentum cuts across the propellerlike structure shown in Figs. S2g, h (taken along the dashed lines in Figs. S2e, f, respectively): the deep electron band giving rise to the large circular feature at the M point (indicated with the white arrow in Fig. S2g) vanishes entirely in the aged sample, whereas the bands contributing to the propellerlike structure remain unaffected. It must, therefore, be concluded that the propellerlike construct is a part of the bulk electronic structure, while the deep electron band producing the large circular feature in Fig. S2e must have a surface-related character.

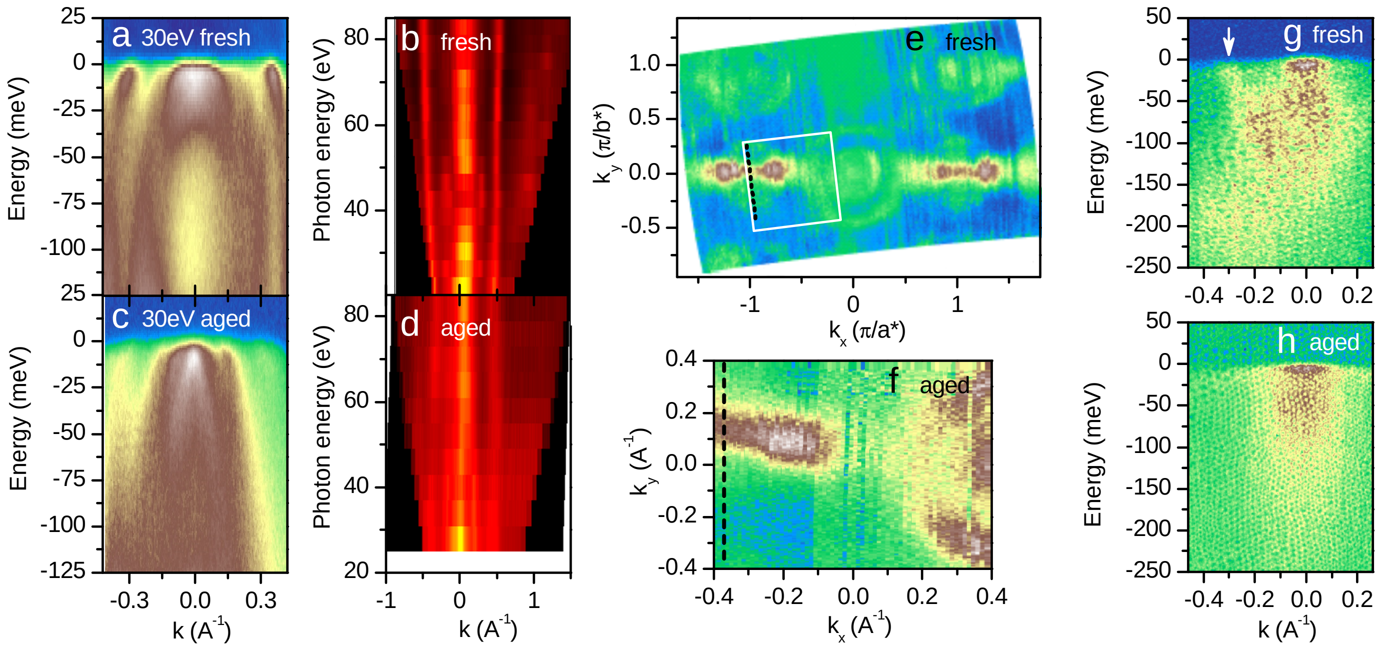

Figure S2 | Effect of surface aging on the low-energy electronic structure of $SmFe_{0.92}Co_{0.08}AsO$ a,c, Band dispersions in the vicinity of the Γ point obtained using incident radiation polarized within the plane of incidence (horizontal polarization) before (**a**) and after (**c**) aging, respectively. **b,d** Dependence of the photoemission intensity in the vicinity of the Fermi level along the Γ-M high-symmetry direction on the energy of the incident radiation, revealing the k_z dispersion of the holelike bands near the Γ point, before and after aging, respectively. **e,** Constant-energy map obtained on the fresh sample, from Fig.1b of the main text. **f,** The same for the aged sample in a narrow momentum region, indicated with a white rectangle in panel **e**. This constant-energy map was obtained by integrating the photoemission intensity in a small energy window of 10 meV around the Fermi level recorded at $T = 1$ K using photons with an excitation energy of 35 eV linearly polarized perpendicular to the plane of incidence. **g,h** Energy-momentum cuts across the propellerlike structure along the black dashed lines in panels **e,f** before and after aging, respectively. The white arrow in panel **g** indicates the deep electron band giving rise to the large circular feature at the M point in panel **e**.

III. ANALYSIS OF THE SURFACE-RELATED ELECTRON BAND AT THE M POINT

We now turn to the detailed analysis of the deep surface-related electron band producing the large circular intensity distribution at the M point in Fig. S2g. Figure S3a shows an energy-momentum cut along the left edge of the white rectangle in Fig. S2g. A very shallow and a much deeper electron band producing the localized intensity spot and a large circular feature near the Fermi level, respectively, are clearly visible. In order to determine the location of the bottom of the deep surface-related electron band, we have taken the second derivative of the experimental data in Fig. S3a with respect to momentum, shown in Fig. S3b. The analysis of the energy-distribution curve averaged within a finite momentum window (indicated by a white rectangle in Fig. S3b) in Fig. S3c indicates that the band bottom is located near 135 meV below the Fermi level.

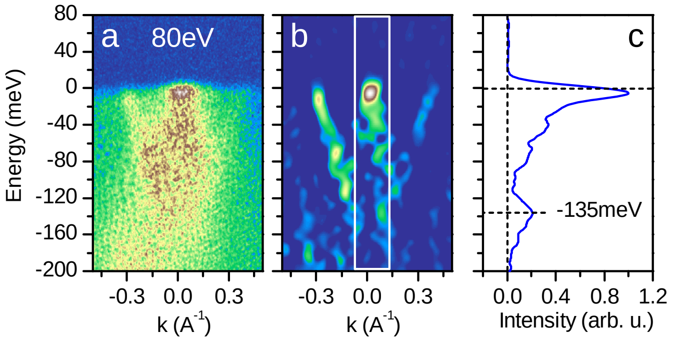

Figure S3 | Low-energy electronic structure of $SmFe_{0.92}Co_{0.08}AsO$ at the M point. a, Energy-momentum cut at the M point perpendicular to the Γ–M direction obtained using photons with an excitation energy of 80 eV linearly polarized perpendicular to the plane of incidence (vertical polarization). **b,** Second derivative of the data in **a** with respect to momentum. **c,** Energy-distribution curve averaged in the momentum window shown as a white rectangle in panel **b**. Horizontal dashed lines indicate the location of the Fermi energy and the bottom of the deep electron band evident in panels **a,b**.

IV. SEPARATION OF THE SURFACE-RELATED BANDS FROM THE BULK BASED ON THE CHARACTERISTIC PROPERTIES OF THE ELECTRONIC STRUCTURE

In this section we would like to utilize the characteristic properties of the electronic band structure, well-established from *ab initio* density-functional calculations on iron pnictides in general and the 1111-type materials in particular, to prove the surface-related character of the second outermost band at the Γ point and shed further light onto the low energy bulk electronic structure in the same region of the Brillouin zone. First of all, based on the electronic structure shown in Fig.1i of the main text, we would like to point

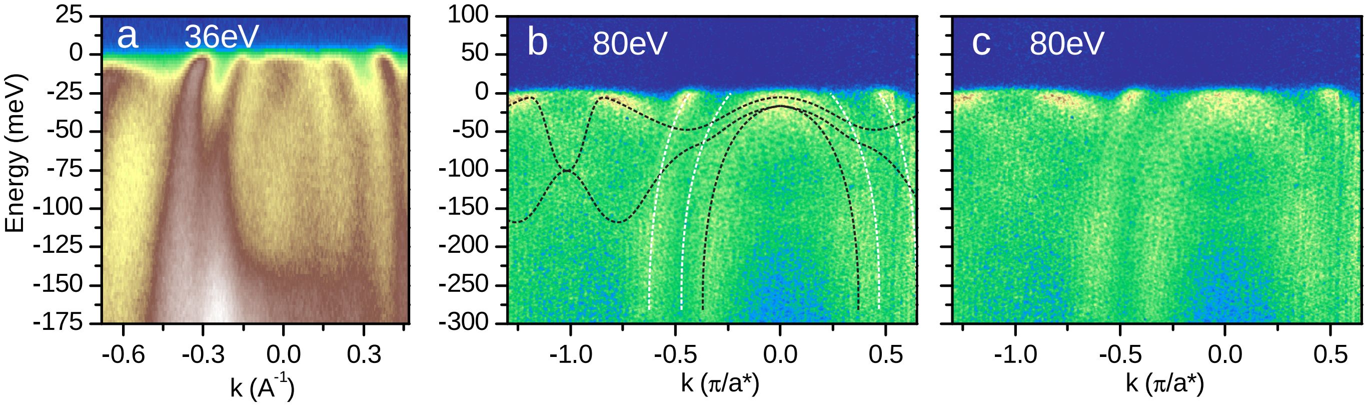

Figure S4 | Continuity of the low-energy electronic structure in $SmFe_{0.92}Co_{0.08}AsO$. a, Energy-momentum cut from Fig. S2b in a larger energy and momentum window. **b,** Energy-momentum cut along the Γ–M direction from the data in Fig.1b of the main text. Black dashed lines represent schematically the bulk electronic band structure expected from the *ab initio* calculation shown in Fig.1i of the main text. White dashed lines indicate the surface-related bands. **c,** Same as panel **b** but without dashed lines to demonstrate the features in the raw data.

out that irrespective of the assignment of various bands observed in the experiment to the surface or bulk, several conditions must

be met: a) the heavy hole band at the M point must connect to one of the three bands at the Γ point closest to the Fermi level; b) the bottom of the deep electron pocket at the M point must connect to another one of the three bands at the Γ point closest to the Fermi level; c) the third band of the three closest to the Fermi level does not connect to the propellerlike construct at the M point and disperses towards higher binding energies in the limited energy window shown in Fig.1i of the main text; d) the deeper two of the three aforementioned bands at the Γ point are quasi-degenerate at the center of the Brillouin zone, the other one is split off them by spin-orbit coupling.

Based on these observations we now show that neither of the two outermost bands at the Γ point can belong to the same band structure as the inner hole bands terminating in the immediate vicinity of the Fermi level and the propellerlike construct at the M point. Figure S4a shows that the two outer bands at the Γ point disperse quasi-quadratically towards high binding energies without signs of rounding off towards the edges of the Brillouin zone, whereas the heavy hole band at the M point (visible in the top left corner of Fig. S4a and, more clearly, in Figs. S4b,c) connects to one of the bands at Γ within 50 meV below the Fermi level. Given that the propellerlike structure is not affected by aging (as opposed to the deep electron band giving rise to the large circular feature at the M point in Fig. S2g, which is clearly suppressed by aging) one must conclude that all the bands producing large circular features in Fig.1a,b of the main text are surface-related. The only remaining issue is the lack of the third hole band in the low energy electronic structure at the Γ point, expected from the *ab initio* calculations (see Fig.1i of the main text). Indeed, up to now we have only shown the existence of two hole bands terminating in the immediate vicinity of the Fermi level (see Fig.1e and Fig. S1), with a splitting of about 25 meV. Quite interestingly, the same analysis as above allows one to maintain that our data are, in fact, consistent with the presence of all three hole bands at the Γ point, shown in Fig.1i of the main text. This can be seen as follows: the top bulk hole band at the Γ point must connect to the heavy hole band at the M point. The splitting between the other two hole bands at the Γ point due to the spin-orbit coupling is expected to be quite small (see Fig. S1c) and could not be resolved in our measurements. However, one of them must connect to the bottom of the propellerlike structure at M within about 150–175 meV below the Fermi level and thus must round off and cannot continue dispersing to higher binding energies. The only of the considered here three hole bands at the Γ point that can is the innermost hole band, as can be seen in Fig.1i of the main text. Figures S4b,c clearly show the presence of a hole band dispersing quasi-quadratically down to at least 300 meV. One must, therefore, conclude that all three hole bands predicted by ab inito calculations contribute to the photoemission intensity near the Γ point.

V. SUPERCONDUCTING ENERGY GAP ON THE SURFACE-RELATED PHOTOEMISSION FEATURES NEAR THE Γ POINT

In order to investigate the effect of superconductivity on the surface-related features (the two outer band dispersions) near the Γ point, we have studied the temperature dependence of these features deep in the superconducting and normal state at an energy of the incident radiation of 30 eV. The results of these measurements are presented in Fig. S5 in the form of energy-distribution curves (EDC) at two characteristic temperatures: in the normal state at 21 K (magenta lines) and the superconducting state at 1 K (black lines). The EDCs have been integrated over a finite momentum range (indicated schematically with black lines in the insets of the respective panels). Both EDCs show a very clear shift of the leading edge on the order of 1.5 meV indicating the presence of a superconducting energy gap in the corresponding electronic dispersions. Since the surface-related bands shown in Fig. S5 do not exhibit any coherence peak in the superconducting state, the magnitude of the superconducting energy gap cannot be extracted reliably through fitting. Therefore, only the leading-edge shift is indicated in the panels as the lower bound of the superconducting energy gap on these bands.

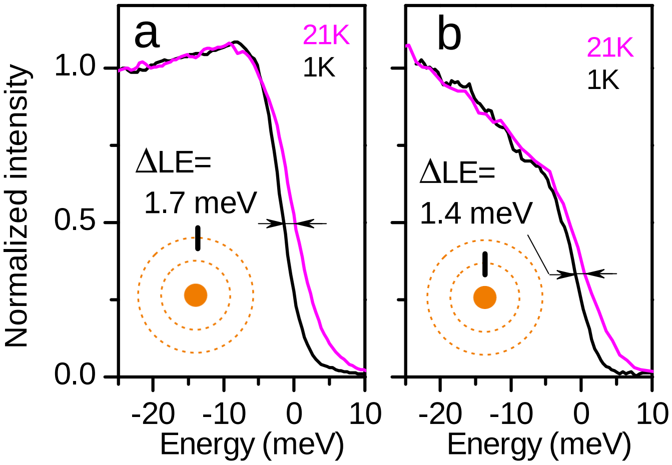

Figure S5 | Superconducting energy gap on the surface-related bands of $SmFe_{0.92}Co_{0.08}AsO$. a,b, Energy-distribution curves in the surface-related electronic structure in the superconducting (1 K) and normal (21 K) state obtained at an energy of the incident radiation of 30 eV and integrated over a finite momentum range as shown by thick black lines in the respective insets. The shift of the leading edge indicates the presence of a superconducting energy gap at low temperatures.
